# Assessment of *Bacillus subtilis* and *Bacillus licheniformis* as Agents Against External Sulfate Attack on Cementitious Materials

**DOI:** 10.3390/ma19112386

**Published:** 2026-06-03

**Authors:** Jonathan Gallardo-Figueroa, Angela Plaza-Garrido, Alvaro Paul, Ivan Navarrete, Leonardo Brescia-Norambuena

**Affiliations:** 1 Departamento de Ingeniería Civil en Obras Civiles, Facultad de Ingeniería, Universidad de Santiago de Chile, Víctor Jara 3659, Santiago 9170124, Chile; jonathan.gallardo@usach.cl (J.G.-F.); angela.plaza@usach.cl (A.P.-G.); 2 Facultad de Ingeniería y Ciencias Aplicadas, Universidad de los Andes, Chile, Mons, Álvaro del Portillo 12455, Las Condes, Santiago 7620001, Chile; apaul@miuandes.cl; 3 Department of Construction Engineering and Management, School of Engineering, Pontificia Universidad Catolica de Chile, Vicuña Mackenna 4860, Macúl, Santiago 7820436, Chile; iinavarr@uc.cl; 4 Concrete Innovation Hub UC (CIHUC), Pontificia Universidad Católica de Chile, Vicuña Mackenna 4860, Macúl, Santiago 7820436, Chile

**Keywords:** sulfate-reducing bacteria in concrete, bioconcrete durability, external sulfate attack, bioconcrete performance

## Abstract

**Highlights:**

- *Bacillus subtilis* and *Bacillus licheniformis* increase the compressive strength of cementitious mixes; however, they are susceptive to overdosage, water–cement ratio, and mix homogenization.
- *Bacillus licheniformis* is highly effective to control mortar expansion by sulfate attack.
- *Bacillus licheniformis* creates changes in mixes’ microstructure that control external sulfate attack better than calcium precipitation.
- Calcium precipitation is effective in terms of strength, but not in terms of preventing deterioration by sulfate attack in the long term.

**Abstract:**

Bacteria in concrete has been studied as an additive to repair microcracks and reduce permeability, as well as increase compressive strength. Within the broad spectrum of bacteria, two types promise to be effective agents against external sulfate attack: (i) *Bacillus subtilis*, which could indirectly prevent the entry of sulfates through the mechanism of sealing by calcium precipitation, and (ii) *Bacillus licheniformis*, which could encapsulate the sulfates that enter by diffusion and prevent the consequences of the pathology, such as expansion and loss of strength. This research evaluates the impact of *B. subtilis* and *B. licheniformis* on the performance of cementitious mixes against external sulfate attack, measuring compressive strength, expansion, permeability, and effects on the microstructure. Results show that both bacteria can produce compressive strength improvements of up to 20% at 28 days and 50% at 180 days. Moreover, in the presence of sulfates, improvements of up to 90% can be observed over control mixes. However, this result should be carefully evaluated because although *B. licheniformis* produces better results in the long term, it results in lower strength in the presence of sulfates in the short term. At the same time, *B. licheniformis* significantly reduces expansion against external sulfate attack, decreasing it by up to 80%, because it generates less ettringite and gypsum. Thus, *B. licheniformis* is an effective agent against external sulfate attack. Based on the results, it is estimated that both bacteria can be used to improve performance; however, care must be taken with concentration, which affects homogeneity or generates negative effects. In particular, it is noteworthy that calcium carbonate loss was observed from the mixes due to continuous curing and that calcium precipitation can generate negative effects against sulfates in the long term.

## 1. Introduction

Portland cement concrete (PCC) is one of the most widely used materials due to its cost/performance ratio [1]. However, throughout its service life, it is subject to various physicochemical processes of deterioration that reduce its durability and sustainability [2]. Among the main mechanisms of deterioration is sulfate attack [3], which affects its mechanical strength, physical integrity, and microstructure. Sulfate attack can be triggered by three different mechanisms [4,5]: (i) internal attack by a high concentration of sulfates inside the PCC’s raw materials, (ii) a high temperature while curing the PCC, generating delayed ettringite formation, or (iii) the penetration of external sulfates present in the exposure medium that react with the microstructure of the hydrated cement paste. Given the severity of sulfate attack, both raw materials and curing temperature are highly controlled to prevent internal attack and attack from delayed ettringite. Nevertheless, external sulfate attack (ESA) is often a concern because it depends on external conditions that are not always controllable. For example, high concentrations of sulfates can be found in groundwater, affecting tunnel and bridge strains in marine areas and piles of concrete structures [6,7]. Consequently, 60% of cases of deterioration due to sulfates correspond to ESA [7]. ESA can be described as an interaction between sulfate ions around concrete that reach the interior of the concrete through diffusion processes [8]; upon entering, these ions react with the hydration products (mainly alumina or monosulfates) of the cement paste, thereby forming ettringite or gypsum in the already hardened paste, which induces expansion, increases permeability, and reduces structural strength [3,9,10,11]. Ettringite is the primary mineral responsible for the expansion of and internal stresses in PCC due to the pressure of new crystallizations generated by the supersaturation of the pores with sulfate [12]. This triggers microcracking when the tensile strength of the PCC’s binder matrix is exceeded; as a result, structural capacity is lost [13]. These expansions, which reach values greater than 1%, can reduce the mechanical strength by up to 60% [14]. At the same time, sulfate attack affects other microstructures of the cement paste; among these cases, gypsum formation is observed due to the reaction of calcium hydroxide with external sulfates, which restarts the reaction cycle, with the monosulfates forming more ettringite [15] and increasing the expansion rate of the concrete [16]. This expansion due to the increase in gypsum inside the cementitious matrix is responsible for 20% of the total expansion due to ESA [17], resulting in a strength loss of up to 70% after 24 months due to the degradation of the microstructure [18].

At present, there are several approaches preventing ESA. Traditional solutions include reducing the water-to-cement (*w*/*c*) ratio [19,20], limiting the amount of C_3_A in cement [21], increasing coating, and reducing permeability with more cement paste [11]. Today, these alternatives have been replaced by more efficient solutions, such as the use of supplementary cementitious materials, including fly ash, slag, silica fume, and metakaolin [22], which are used to partially replace the cement and which densify the microstructure by favoring the formation of hydrated calcium silicate from the pozzolanic reaction [23,24]. However, limitations, such as greater logistical management of raw materials, local availability, and market shortages, are increasingly affecting their implementation [25]. Because of the above situation, a new approach focused on biotechnologies for concrete has emerged in recent years, giving rise to bioconcretes.

Bioconcretes use bacteria to improve concrete performance, focusing on sealing microcracks through calcium precipitation [26,27] concrete because this favors the entry of chemical agents from the outside. According to Wiktor and Jonkers [26], the calcium precipitation effect improves resistance to chemical attacks by recovering permeability to ranges close to those pre-cracking. Bioconcretes can also improve healthy concretes by filling natural micropores [28]. However, this effect is delayed in comparison with that of self-healing [28].

Bacteria use is complex because the survival of bacteria during mixing is essential to improving the performance of the concrete [29,30]. For this reason, about 84% of studies on the use of bacteria in concrete have focused on the *Bacillus* group, as one of the main advantages of *Bacillus* is that they can sporulate, which makes them more resistant to the mixing process [31]. This sporulation, which is the replication mechanism of bacteria, generates spores that survive for more than 50 years in adverse conditions, such as high temperatures and highly alkaline environments [32]. In this way, the spore remains inactive in the concrete until it encounters conditions that allow it to be fully or partially activated, such as contact with water entering through the microcracks, which generates calcium precipitation [33]. An example of this is the application of *Bacillus subtilis*, which increased compressive strength, reaching improvements of up to 27% with a concentration of 10^5^ cells/mL, and improved permeability, reducing water absorption by 27% with a concentration of 10^7^ cells/mL by sealing all microcracks with thicknesses up to 1.2 mm, at 28 days of curing [28]. Other studies indicate improvements in compressive strength of 15% and 23% with a concentration of 0.8 × 10^8^ cells/mL of *B. subtilis* and *B. megateria*, respectively [34]. Basha et al [35] obtained improvements in compressive strength of 28%, 20%, 16%, and 13% at concentrations of 10^4^ (cells/mL) of *B. subtilis*, *Paenibacillus dentritiformis*, *B. methylotrophicus*, and *B. licheniformis*, respectively. Chahal et al. [36] obtained compressive strength improvements of up to 22% after the application of the bacterium *Sporoscarcina pasteurii* in combination with fly ash with a bacteria concentration of 10^5^ (cells/mL). Next, Table 1 is a summary of different bacteria studies on concrete is shown to enable understanding of the current concentration framework.

Among these bacteria, *B. subtilis* stands out for its ease of use and for being a non-pathogenic bacterium [49] common in the gut microbiota and soil [50]. Another remarkable bacterium is *B. licheniformis*, which is also non-pathogenic and used in multiple applications in medicine and industry [50]. However, unlike *B. subtilis*, *B. licheniformis* is a sulforeductive bacterium that, in addition to precipitating calcium, has metabolic pathways capable of reducing sulfates in the environment [51,52]. According to Rückert (2016) [53], sulfates are reduced mainly through two pathways: dissimilative reduction (DSR) and assimilative reduction (ASR). In both cases, the reduction involves the production of sulfites and sulfides, which could affect concrete through corrosion, but the ASR pathway reduces these products used by the bacterium for its biological processes, transforming them into a harmless protein called L-cysteine. Thus, if *B. licheniformis* reduces sulfate through the ASR pathway, then it could provide a sulfate encapsulation mechanism. However, this finding has not yet been evaluated in concrete, highlighting two gaps to be evaluated: (i) How does the bacteria metabolic pathway affect the durability of concrete? (ii) How does calcium precipitation interact with ESA?

Consequently, this is one of the first studies addressing the application of bacteria in cement pastes subjected to external sulfate attack (ESA), particularly evaluating the potential benefits of bacterial sulforeductive pathways rather than focusing only on calcium precipitation mechanisms. *B. subtilis* was used as a net calcium-precipitating bacterium and *B. licheniformis* was employed to evaluate the bacterial sulforeductive capacity at concentrations of 10^5^, 10^6^, and 10^7^ (cells/mL) in mixes with *w*/*c* ratios of 0.3 and 0.5, representing low- and medium-permeability mixes, respectively [52,54]. ESA was induced by curing the specimens in sodium sulfate, which, according to Whittaker and Black (2015) [9], is a highly soluble salt typically used in tests such as ASTM C1012 [55]. The compressive strength was evaluated on 50 × 50 × 50 mm cubes at 7, 28, 56, 90, and 180 days, both for mixes cured in water and in sulfates. ESA expansion in mortar was also tested between 1 and 17 weeks. To complement the performance and microstructural changes, surface electrical resistivity, X-ray diffraction (XRD), thermogravimetry analysis (TGA), infrared spectroscopy (FTIR), and scanning electron microscopy (SEM) were implemented. The results showed that *B. subtilis* and *B. licheniformis* can improve the performance of cementitious mixes both in water and under external sulfate attack. Under water curing, both bacteria increased compressive strength, reaching gains of up to 20% at 28 days and up to 50% at 180 days, depending on mix design. However, the results of mixes exposed to sodium sulfate are more interesting: in particular, *B. licheniformis* achieved a high strength increase with a simultaneous reduction in mortar expansion; in contrast, *B. subtilis* showed a tendency toward higher expansion and progressive long-term instability at elevated concentrations. XRD analyses revealed that sulfate-cured *B. subtilis* mixtures increased ettringite and gypsum contents by approximately 15% and 6%, respectively, whereas *B. licheniformis* reduced these phases by about 7% and 4%. FTIR spectra supported these observations through attenuation of sulfate-related bands (1100–1200 cm^−1^) and preservation of C–S–H-associated bands (~950–1055 cm^−1^) in *B. licheniformis* mixtures. TGA showed changes in carbonate-associated phases while limiting sulfate-related transformations. Despite these chemical and mechanical differences, surface electrical resistivity remained relatively unchanged, suggesting that the bacterial contribution was primarily governed by chemo-mineralogical modifications rather than generalized permeability reduction. Collectively, the results indicate that *B. licheniformis* may alter ESA response beyond a simple pore-blocking system.

## 2. Materials and Methods

### 2.1. Materials

**Figure 1:**
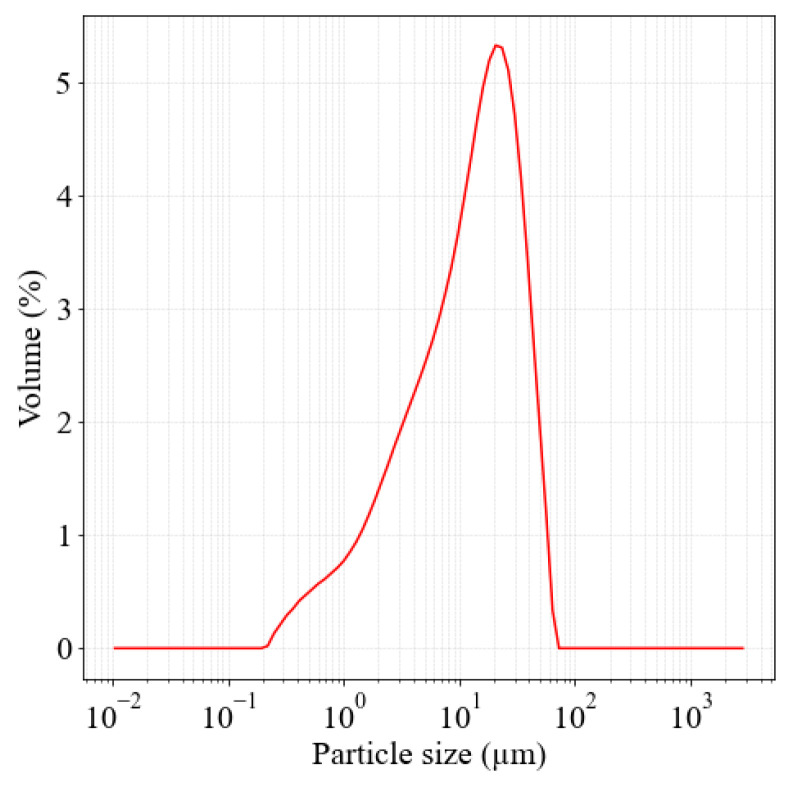
Cement particle size distribution.

For compressive strength tests and chemical analysis, cement paste mixes were prepared. Mortar mixes were made as explained below and used to measure expansion due to sulfate attack. The cement pastes and mortar mixes were made using Type I cement according to the ASTM C150 (Melón Super cement, Santiago, Chile [56]). Table 2 shows a cement X-ray fluorescence (XRF) analysis, and cement particle size is shown in Figure 1, using a Malvern Mastersizer 2000 laser diffraction analyzer (Malvern Instruments Ltd., Malvern, UK). Mortars were made with normalized natural silica sand and monoparticle sand N°30 (92%) according to ASTM C778 [57], with a density of 2592 kg/m^3^ and absorption of 0.8%. This sand was chosen to minimize aggregate-related variability during external sulfate attack exposure without affecting the mortars’ workability. The workability of the mix was adjusted by using Viscocrete 6000 high-range water-reducing admixture, which has a density of 1050 g/L and was added in doses of 0.1–0.3% of the cement weight according to the *w*/*c* ratio.

*B. subtilis* and *B. licheniformis* were obtained in a dry state from the DSMZ repository and then cultured in liquid medium (Medium1) consisting of distilled water, peptone (5 g/L), and meat extract (3 g/L). A total of 20 mL of the medium with bacteria was obtained and seeded in plates with Medium1, agar (15 g/L), and MnSO_4_xH_2_O (10 mg/L) for sporulation. After 72 h, the plates were washed with distilled water to rescue the spores, and the recovered material was centrifuged at 4000 rpm three times to separate and wash the obtained spores. Finally, the spores were diluted in 50 mL of distilled water to obtain the desired concentration in each mix. The experimental matrix, which is shown in Table 3, considered three concentrations of bacteria—10^5^, 10^6^, and 10^7^ cells/mL—together with the control mixes that did not contain bacteria. In addition, *w*/*c* ratios of 0.3 and 0.5 were considered for the experimental mix design. The mortar specimens were a limited version of cement paste specimens, considering the limit concentrations of bacteria. For the cement paste mixes, 50 × 50 × 50 mm cubic specimens were made to measure compressive strength according to ASTM C109 [58], and 100 × 200 mm cylinders were made to measure surface electrical resistivity according to AASHTO T358 [59]. A mortar prism with dimensions of 25 × 25 × 285 mm was also made to measure sulfate expansion based on ASTM C1012 [55] with a constant sand–paste ratio, following the ratio established by the ASTM.

### 2.2. Mixing Procedure and Sample Preparation

For the mixing process, the bacteria were pre-dosed to reach the final concentration according to the experimental matrix in 50 mL of water, i.e., the total water in the mix was multiplied by the bacteria concentration. Then, 50 mL of water–bacteria mix was homogenized in an ultrasonic bath for 300 s, directly added to water with high-range water-reducing admixture and mixed for 15 s until the liquid materials were homogenized. Following this, the cement was added and mixed for 30 s on low speed, and unmixed material from the bottom of the mixer and the edges was manually placed in the mix in less than 15 s. Finally, the paste was mixed at high speed for 90 s. After the mixing process, the cement paste was poured into the cubic specimens and cylinders, using a vibrating table to compact the fresh mix, and then covered with film to prevent water loss. After 24 h, the samples were unmolded and immersed in water. Some of the cubic specimens were immersed in water with sodium sulfate with a concentration of 50 g/L after 14 days to induce sulfate attack based on the ASTM C1012 exposure definition for length change evaluation. The mortar mixing procedure was similar to the procedure for creating cement pastes. After the cement paste was homogenized, sand was added and mixed for 30 s at low speed. Finally, without stopping, mixing was carried out at medium speed for 1 more minute. The mortar mixes were designed to maintain the cement paste–sand ratio recommended for ASTM C1012, but using the *w*/*c* ratio of the experimental matrix. The 0.3 and 0.5 *w/c* ratios were selected to represent low- and medium-permeability concretes [52,54].

### 2.3. Mechanical and Durability Testing

The cement pastes’ compressive strength was measured with three cubic specimens of 50 × 50 × 50 mm at 7, 28, 56, 90, and 180 days according to the ASTM C109 test, which was carried out with the Tecnotest machine (model KE300/CE, Tecnotest, Modena, Italy), which has a maximum capacity of 3000 kN. Surface electrical resistivity was checked using a Proceq Resipod (Proceq SA, Schwerzenbach, Switzerland) in accordance with the AASTHO TT358 standard [59], measured at 7, 28, 56, and 90 days. The test included two rounds of measurements every 90°, with three 100 × 200 mm cylinders saturated in water. The sulfate attack expansion test was performed by measuring a 25 × 25 × 285 mm mortar prism using the Controls with 62-L0035/A (CONTROLS Group, Milan, Italy) equipment based on the ASTM C1012 standard. The length of the beams was measured at 1, 2, 3, 4, 5, 6, 7, 12, 15, and 17 weeks, and 3 beams were measured per mix at each age.

### 2.4. Microstructure Characterization

For the analysis of the microstructure, XRD, TGA, FTIR, and SEM were performed on the control cement paste mix, and mixes 6 and 12. Samples for XRD, TGA, and FTIR were measured in powder format, while those with granular pieces smaller than 2 mm were used for SEM. In all cases, the samples were obtained from the cores of the cubes that were tested under compression. An age of 90 days was selected because results showed a relative stabilization of strength and expansion behavior.

XRD was performed at 28 days to determine the mineral composition of the samples by using a Bruker D2 Phaser diffractometer (Bruker, Billerica, MA, USA) at 30 kV/10 mA, with a range of 10° to 90° and a step size of 0.01 to 1.25 s.

TGA was done at 28 and 90 days using Q600 from TA Instruments (TA Instruments, New Castle, DE, USA), with a temperature ramp of 20 °C/min and a flow of 50 mL/min of nitrogen until a temperature of 1000 °C was reached.

Fourier transform infrared (FTIR) analysis was conducted with a Shimadzu IRTracer-100 (Shimadzu Corporation, Kyoto, Japan) with a diffuse reflectance module, measuring between 400 and 4000 cm^−1^.

The morphology of the cement paste was analyzed by scanning field-emission SEM with energy-dispersive X-ray spectroscopy (EDX) with a 2 nm gold coating. A Quanta FEG250 microscope was used to obtain high-resolution images.

### 2.5. Statistical Analysis

Finally, statistical analysis of the compressive strength tests was carried out using the normalized β-coefficients of Equation (1) in an ordinary least squares model, shown below, to obtain a physical representation of the experimental variables’ weight and nonlinearity. C represents the concentration of bacteria in cells/mL, and *w*/*c* is the water–cement ratio.

(1) $$fc\;=\beta 0\;+\beta 1\cdot \frac{w}{c}+\beta 2\cdot \;C+\beta 3\;\cdot C^{2}+\beta 4\cdot \;t+\beta 5\cdot \;t^{2}+\varepsilon$$

## 3. Results

### 3.1. Compressive Strength

**Figure 2:**
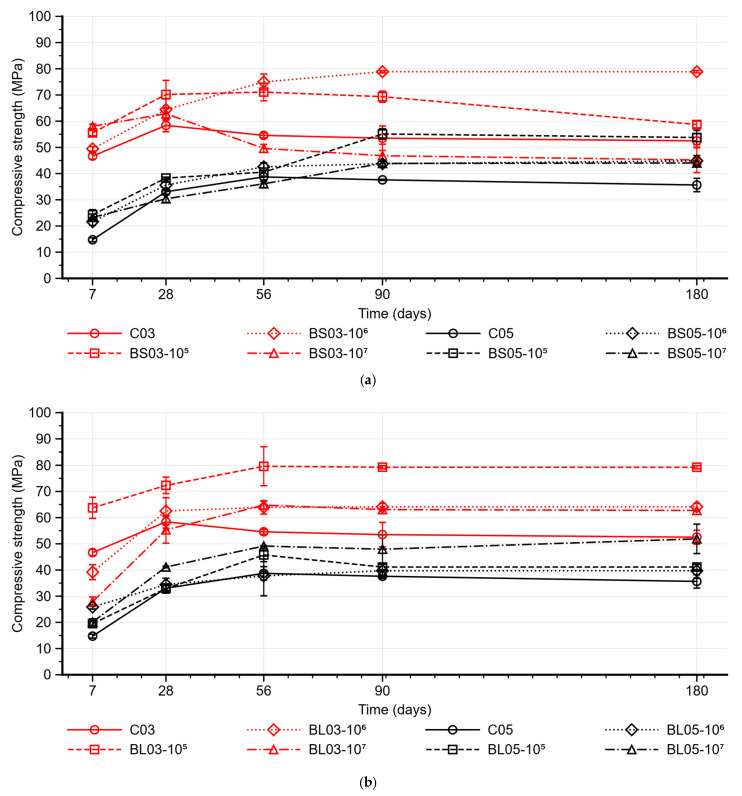
Compressive strength of water-cured cubes with (**a**) *B. subtilis* and (**b**) *B. licheniformis*.

Figure 2 shows the compressive strength of cement pastes with water-cured *B. subtilis* and *B. licheniformis*. In mixes with *w*/*c* = 0.3, the incorporation of *B. subtilis* generates an increase in strength at an early age, which decreases over the curing time compared with the control mix. In particular, at 7 days, the mixes show an average increase of 16% compared with the control mix, highlighting the concentration of 10^7^ cells/mL, which reaches an increase of close to 24%. This behavior is consistent with the early precipitation of calcium carbonate, which densifies the matrix in the initial stages of hydration. At 28 days, the positive effect is intensified for the 10^5^ and 10^6^ concentrations, which show increases in the order of 10–20%, while the 10^7^ dose shows a more moderate benefit. At 56 and 90 days, more varied behavior is observed depending on the concentration: the 10^6^ concentration is the most efficient, reaching increases of up to 47% compared with the control, while the 10^7^ dose shows a progressive loss of strength. These values are maintained up to 180 days in approximate terms. In pastes with *w*/*c* = 0.5, the effect is even more pronounced, explained by the expected differences in porosity and strength between mixes with *w*/*c* = 0.5 and *w*/*c* = 0.3. Consequently, at 7 days, increases of up to 65% are observed with respect to the control mix, which decrease at 28 and 56 days to a range of 15–35%, with the 10^5^ concentration presenting the most stable behavior. However, after 90 days, all mixes with *B. subtilis* converge toward similar values, and at 180 days, the differences with respect to the control are reduced to moderate increments of 20–30%. This result can be explained on the basis that high doses of bacteria can demonstrate early strength by sealing pores with calcium carbonate; however, in the long term, given the solubility of calcium carbonate in water, which generates Ca^+^ and CO_2_, strength can be lost. The dissolution of carbonate phases in aqueous medium has been previously reported as responsible for lost strength and microstructural changes [60,61]. Although low doses do not generate this loss in the long term, they are not effective enough to densify and maintain the advantage over control mixes. In addition, although bacterial activity favors strength, excessive concentrations can generate negative effects, such as microheterogeneities or localized increases in porosity.

For pastes with *w*/*c* = 0.3, *B. licheniformis* exhibits a different behavior than *B. subtilis*. At 7 days, high dispersion is observed, and low doses of bacteria (10^5^) increase the strength by approximately 37%, whereas higher doses generate a progressive loss of strength. However, from 28 days onward, the concentration of 10^5^ generates a relatively constant strength, which is always above that of the control mix. Concentrations of 10^6^ and 10^7^ show increasing strength over time, surpassing the control mix after 56 days. In mixes with *w*/*c* = 0.5, *B. licheniformis* shows a similar effect, where the lowest concentration results in strength above that of the control mix, but the medium and high concentrations require about 28 days, with the highest concentration providing the highest strength at 180 days. In this case, the performance of *B. licheniformis* at low concentrations could be associated not only with calcium carbonate precipitation but also with its metabolic pathways of sulfate reduction, resulting in a decrease in the rate of strength gain either due to the bacteria–internal sulfate interaction or intrinsically slower bacterial activity.

Notably, during curing, a slight decrease in strength is observed even in the control mix. The natural degradation of the microstructure is mainly associated with constant submerged curing [28,62]. Samples of 20 × 20 × 20 mm cubes were made, which, after two months in water, generated a recoverable white material that corresponds to calcium carbonate, as will be explained in the following sections. Although it was not possible to quantify the leachate amount of calcium carbonate due to its uncontrollable adhesion with the surface of the water container, a greater amount is observed in the samples with bacteria. Therefore, if the slope of the strength curves is contrasted in the long term, then it could be estimated that mixes with *B. licheniformis* are more stable during constant water curing than those with *B. subtilis*.

In statistical terms (Table 4), the analysis of *B. subtilis* (R^2^ = 0.78) shows that although the prediction is highly variable, strength is statistically influenced by the *w*/*c* ratio, the concentration of spores per mL, and time. Both concentration and time indicate a quadratic influence, implying in both cases that a higher value of the variable would result in a loss. The *w*/*c* ratio does not interact directly with the other variables. In addition, although adding bacteria can increase strength, the *w*/*c* ratio and time are still the predominant variables. For *B. licheniformis* (R^2^ = 0.82), the concentration is not statistically significant, with the process being mainly controlled by time. However, the results clearly show that the lowest dose of *B. licheniformis* at *w*/*c* = 0.3 and concentrations of 10^5^ and 10^7^ generated an increase in strength over time, which can be observed with a 95% confidence level using a paired T-test.

**Figure 3:**
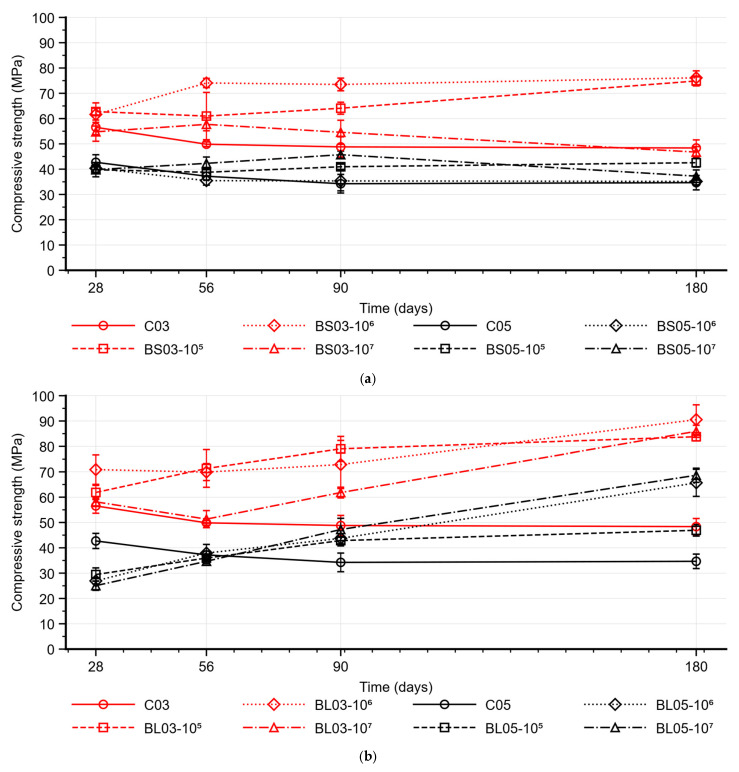
Compressive strength of sulfate-cured cubes with (**a**) *B. subtilis* and (**b**) *B. licheniformis*.

Figure 3 shows the compressive strength of mixes with *B. subtilis* and *B. licheniformis* cured in a sodium sulfate solution at 50 g/L between 28 and 180 days. As can be seen, exposure to sulfates modifies the strength of the pastes until it reaches a significant level of damage. However, this damage is mitigated somewhat by the incorporation of bacteria. In mixes with *w*/*c* = 0.3, the incorporation of *B. subtilis* generates initial improvements in strength compared with the control mix exposed to sulfates. At 28 days, 10^5^ and 10^6^ cell/mL concentrations show increases in the order of 11–27%, while the 10^7^ concentration does not show a significant benefit. At 56 days, the bacterial effect intensifies at the 10^6^ concentration, reaching increases of about 48% compared with the control, suggesting a densification of the microstructure. However, at older ages (90 and 180 days), the 10^7^ concentration presents a progressive loss of strength, reaching values even lower than those of the control mix, indicating that under a high concentration of sulfates, the overdosage of *B. subtilis* may generate a more vulnerable microstructure in the long term, possibly due to the redissolution of calcium carbonate or gypsum formed by the calcium carbonate–sodium sulfate interaction [63]. By contrast, concentrations of 10^5^ and 10^6^ maintain increases in the order of 50–55% at 180 days. In pastes with *w*/*c* = 0.5, the effect of *B. subtilis* is less immediate. At 28 days, the strengths are similar or slightly lower than those of the control mix, suggesting that bacterial precipitation is not sufficient to counteract the initial sulfate attack. However, after 56 days, the 10^5^ concentration begins to show moderate increases, reaching improvements in the order of 6–18%, while higher concentrations show more erratic behavior. At 90 and 180 days, all *B. subtilis* mixes outperform the control mix, with increases reaching up to 55% at 180 days. However, the absence of a clear trend with concentration reinforces the idea that under severe sulfate attack, *B. subtilis* acts primarily as a retarder of deterioration rather than as a sustained mechanical reinforcement, as it is especially sensitive to overdosing.

For pastes with *w*/*c* = 0.3, *B. licheniformis* exhibits markedly different behavior. At 28 days, all bacterial concentrations generate significant increases in strength compared with the control mix exposed to sulfates, highlighting that concentrations of 10^5^ and 10^6^ produce increases close to 25–26%. At 56 days, the effect intensifies, reaching increases of the order of 45–60%, which indicates that sulfate attack is effectively mitigated at relatively early ages. At later ages (90 and 180 days), *B. licheniformis* mixes maintain a strength increase greater than that recorded at 28 days for the different bacteria concentrations, suggesting that *B. licheniformis* not only densifies the matrix by calcium carbonate precipitation but could also chemically interact with sulfates in the medium, reducing their availability to react with cement hydration products. In pastes with *w*/*c* = 0.5, *B. licheniformis* shows progressive and time-dependent behavior. At 28 days, the strengths are similar to or lower than those of the control mix, reflecting the severity of ESA in highly permeable matrices. However, at 56 days, concentrations of 10^5^ and 10^7^ begin to exceed the control, with increases in the order of 18–29%. At 90 and 180 days, the effect becomes greater, particularly for the 10^7^ concentration, which reaches increases close to 98% at 180 days, while the 10^5^ and 10^6^ concentrations show more moderate but sustained improvements. The high strength difference between mixes with bacteria and control mixes is highlighted because *B. licheniformis* tends to increase strength over time, while control mixes reduce it by sulfate attack, suggesting that the bacterium’s metabolic activity favors long-term densification in the presence of sodium sulfate.

From a statistical point of view (Table 5), the OLS model for *B. subtilis* (R^2^ = 0.73) indicates that strength under sulfate attack is influenced by the *w*/*c* ratio and the concentration of spores, with the latter acting quadratically, that is, with nonlinear effects associated with overdosage and progressive deterioration. By contrast, the model for *B. licheniformis* (R^2^ = 0.80) indicates similar behavior dependent on the *w*/*c* ratio and the concentration of bacteria. In the case of *B. subtilis*, time does not indicate major changes, thus losing its statistical relevance regarding the other variables; however, when the control mixes are removed, time exerts a practically linear effect, but given the variability of the results, it reaches *p* = 0.105, leaving a trend, but not strong statistical significance.

### 3.2. Expansion

**Figure 4:**
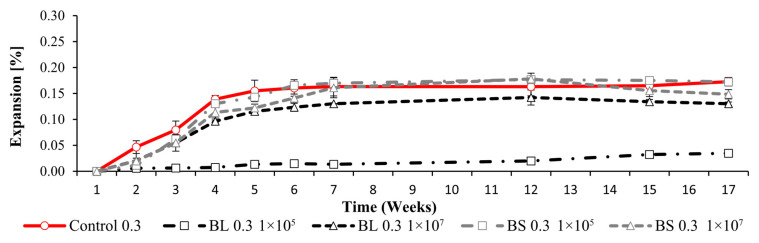
Expansion of 0.3 water–cement ratio mortars exposed to sulfate attack.

**Figure 5:**
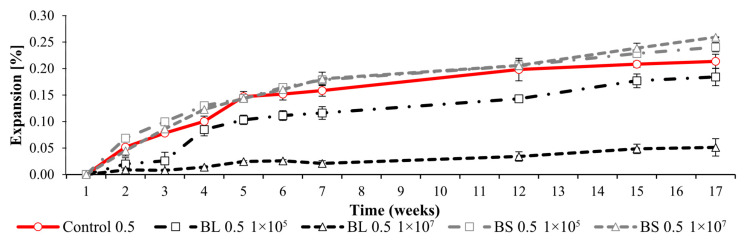
Expansion of 0.5 water–cement ratio mortars exposed to sulfate attack.

The expansion results of sodium-sulfate-cured mortar joists with *w*/*c* = 0.3 and *w*/*c* = 0.5 are shown in Figure 4 and Figure 5, respectively. The application of *B. licheniformis* in mixes with *w*/*c* = 0.3 under sulfate-curing conditions can reduce expansion by up to 80% with concentrations of 10^5^ cells/mL and by around 25% with a concentration of 10^7^ cells/mL. Similarly, for *w*/*c* = 0.5, it reduces expansion by 76% for a concentration of 10^7^ cells/mL and by 15% for 10^5^ cells/mL, indicating that the mixes with a higher *w*/*c* ratio require a higher bacteria dosage to control expansion. In contrast, the application of *B. subtilis* does not reduce expansion; as a matter of fact, *B. subtilis* slightly increases it. Its application at both concentrations shows a tendency to increase expansion by 17% compared to the control group, especially with 10^7^ cells/mL, meaning that even though different authors say that *B. subtilis* increases durability, this is stated from the permeability point of view, without any consideration of sulfate attack. In this case, the results show a poor response of *B. subtilis* in the long term. In agreement with the following sections, *B. licheniformis*’s sulforeductive action acts with the incoming sulfates, while *B. subtilis*’s calcium precipitation reacts to the presence of external sulfates, generating expansive products. Thus, it could be inferred that *B. licheniformis* acts more in the chemical reaction that generates mortar expansion than in the pore-blocking mechanism, which had been previously reported in the literature as a more relevant parameter.

### 3.3. Surface Electrical Resistivity

**Figure 6:**
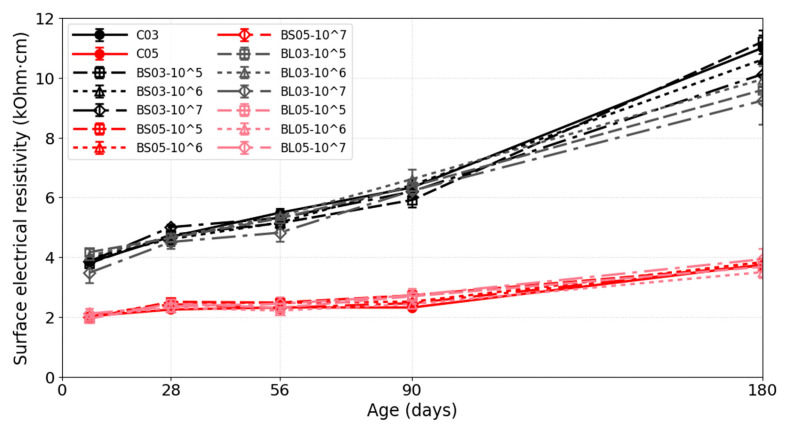
Surface electrical resistivity.

The surface electrical resistivity of mixes with *B. subtilis* and *B. Licheniformis* at 7, 28, 56, and 90 days is shown in Figure 6. In this case, both bacteria have similar levels of resistivity between them and compared with the control mixes. This condition remains constant over time, with less than 10% variation between mixes, indicating two significant results: First, while the literature highlights the permeability reduction by calcium precipitation, the surface electrical resistivity does not capture such an effect. Second, indirect permeability indicates that chemical changes in the pastes created by the bacteria are responsible for the changes in expansion and strength. This result could be validated considering the long exposure of the samples, assuming that all samples started in a healthy condition. It is worth noting that the relatively limited changes in the resistivity and the reduction in expansion support the assumption that sulfate deterioration is dependent on the microstructure phases’ capacity to react with external sulfate instead of on the pore-sealing effect; however, it must be mentioned that surface electrical resistivity mainly reflects ionic migration through the cementitious matrix rather than direct pore connectivity or hydraulic transport.

### 3.4. X-Ray Diffraction

**Figure 7:**
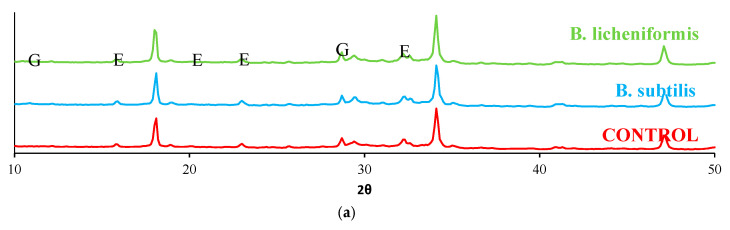

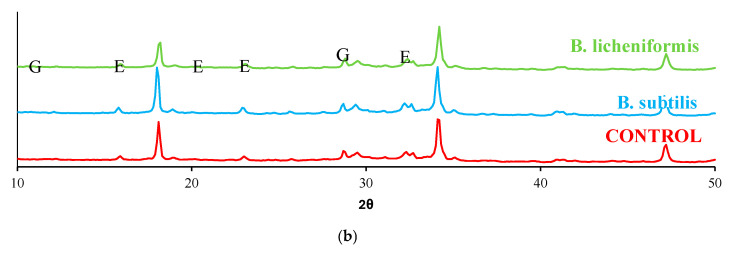
XRD at 28 days cured in (**a**) water and (**b**) sodium sulfate. E: Ettringite; G: Gypsum.

The XRD analysis results for water-cured and sulfate-cured specimens are shown in Figure 7a,b, respectively. The XRD results of 28-day water-cured specimens reveal small differences in ettringite and gypsum production. Following the application of *B. subtilis* and *B. licheniformis*, ettringite peaks increased by an average of 6% and 8%, respectively. The presence of gypsum increased by 5% in the sample with *B. licheniformis*, while for the sample with *B. subtilis*, the value is similar to that of the control sample. Calcium precipitation favors the presence of sulfate-related phases without the need for an external source of sulfate. Notably, the analysis of the sodium-sulfate-cured specimens reveals differences with the water-cured specimens. The presence of ettringite in sulfate-cured samples from the control group and *B. subtilis* group increased by 2% and 15%, respectively; however, it reduced on average by 7% in the sample with *B. licheniformis* after being cured in sodium sulfate. With regard to the presence of gypsum, it was similar in the control mix when cured in water and sulfate, while *B. subtilis* increased it by 6% due to sulfate exposure. By contrast, as with ettringite, the application of *B. licheniformis* decreased the presence of gypsum by 4%, indicating that the precipitation of calcium, in the presence of sulfates, could increase the formation of gypsum, which, in turn, reacts with the aluminate-rich phases present in the microstructure to form more ettringite. Moreover, the sulforeductive pathway could be controlling sulfates through *B. licheniformis*’s ASR pathway. It is worth mentioning that the greater effect of *B. licheniformis* in mixes cured in sodium sulfate may be because the spores require certain environmental conditions for activation of both themselves and the enzymes they release into the cement paste. Therefore, it is possible that the ASR pathway is activated mainly under conditions of severe exposure.

### 3.5. Thermogravimetry Analysis

**Figure 8:**
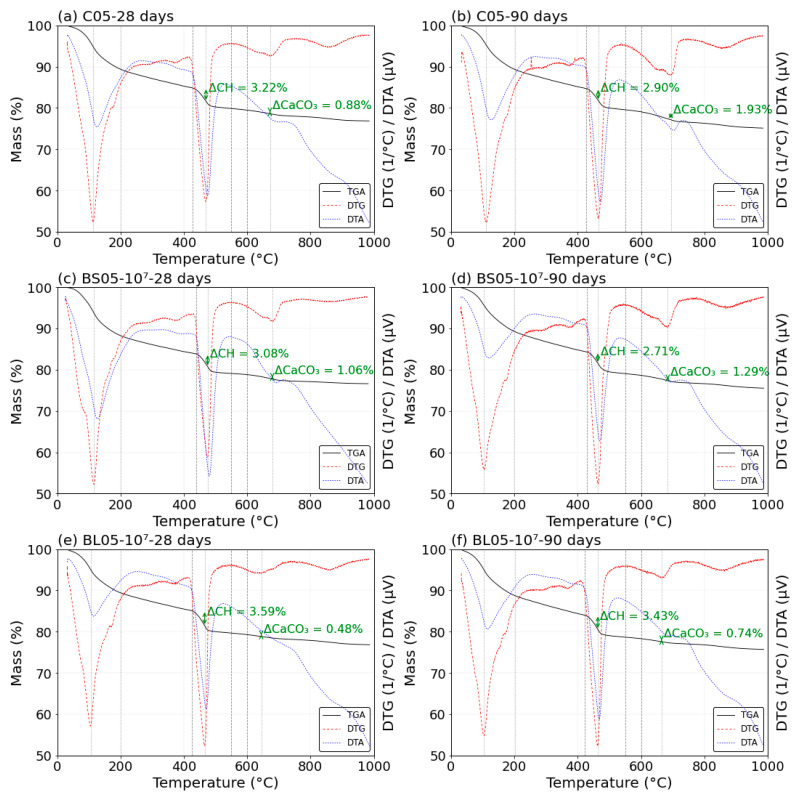
TGA for 0.5 water–cement ratio mixes cured in water: (**a**) control at 28 days, (**b**) control at 90 days, (**c**) BS 10^7^ at 28 days, (**d**) BS 10^7^ at 90 days, (**e**) BL 10^7^ at 28 days, and (**f**) BL 10^7^ at 90 days.

**Figure 9:**
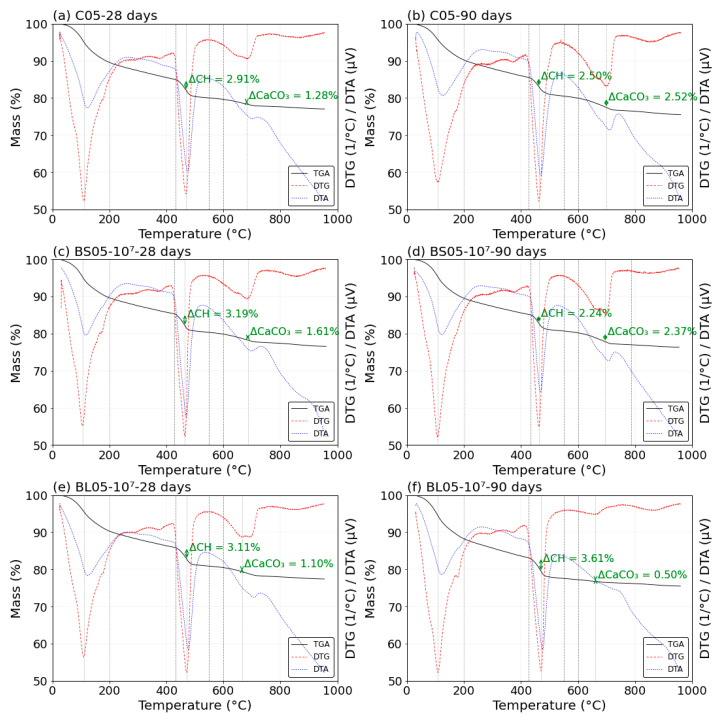
TGA for 0.5 water–cement ratio mixes cured in sulfate: (**a**) control at 28 days, (**b**) control at 90 days, (**c**) BS 10^7^ at 28 days, (**d**) BS 10^7^ at 90 days, (**e**) BL 10^7^ at 28 days, and (**f**) BL 10^7^ at 90 days.

Figure 8 and Figure 9 show the TGA of the mixes with a *w*/*c* ratio of 0.5 at 28 and 90 days and bacteria concentration of 10^7^ cured in water and sodium sulfate, respectively, as they were the mixes most sensitive to changes in strength. Table 6 and Table 7 summarize the main changes evidenced in the DTG curve. At 28 days, the control mix presents typical mass losses associated with hydration products and less stable phases (<200 °C), as well as losses attributable to ettringite and gypsum between approximately 80 °C and 150 °C. Calcium hydroxide breaks down in the temperature range of 400–500 °C, and calcium carbonate is decarbonized in the temperature range of 600 °C and 800 °C. In contrast to these results, *B. subtilis* shows relative increases in mass loss in the range 600–800 °C, consistent with its role as a calcium precipitator, favoring calcium carbonate formation. At 90 days, the mixes present lower mass losses at low temperatures due to a higher level of microstructure densification, and ettringite and gypsum transformation. Considering that mixes with *B. subtilis* and *B. licheniformis* also present minor variations between 28 and 90 days under 150 °C, this suggests either that there is a low demand for ASR activity, or that, although the bacterium controls the presence of sulfate-rich phases, these are encapsulated and released during TGA calcination. Dehydration in the 150–400 °C and 400–550 °C ranges at 90 days suggests that *B. licheniformis* is less reactive with CH than *B. subtilis*; however, the mass loss associated with the 550–800 °C range suggests that the former contributes more to carbonate phases. On the other hand, the specimens cured in sulfates at 28 days show an increase in mass losses in the range 80–150 °C, associated with the formation of ettringite and gypsum, both in the control mix and in the mixes with bacteria. Compared with water curing, mixes with *B. subtilis* show additional increases in the calcium carbonate range, attributable to their calcium precipitation capacity. In *B. licheniformis* mixes, these variations are more marked, which may be related not only to calcium carbonate precipitation but also to the bacterium’s sulforeductive metabolism via the assimilative pathway, which involves the incorporation of reduced sulfate into organic compounds (e.g., L-cysteine), potentially reducing the fraction of free sulfates available for expansive reactions. Then, at 90 days in sulfate, the differences are maintained and, in some ranges, intensified. Carbonate losses are higher than those in water curing, especially in *B. licheniformis* mixes, while losses in the ettringite/gypsum range remain significant. Taken together, these results indicate that the sulfated medium amplifies phase transformations and that the *B. licheniformis* pastes exhibit a differentiated response with respect to *B. subtilis*, consistent with the combination of calcium precipitation and assimilative sulfate reduction.

Table 6 and Table 7 summarize the TGA results.

### 3.6. Fourier Transform Infrared Spectroscopy

**Figure 10:**
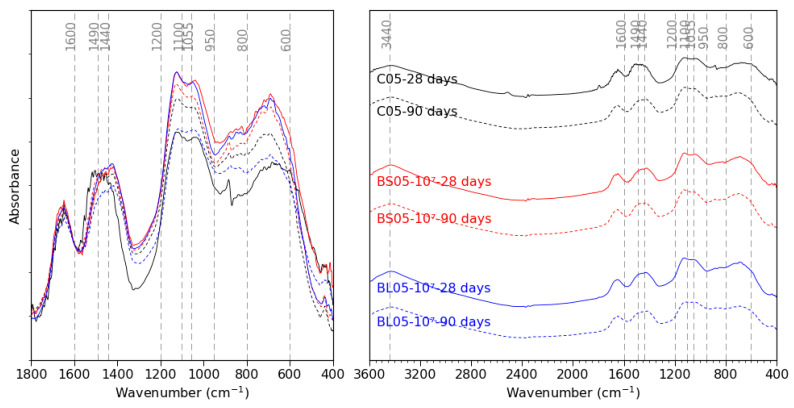
FTIR for 0.5 water–cement ratio mixes cured in water.

**Figure 11:**
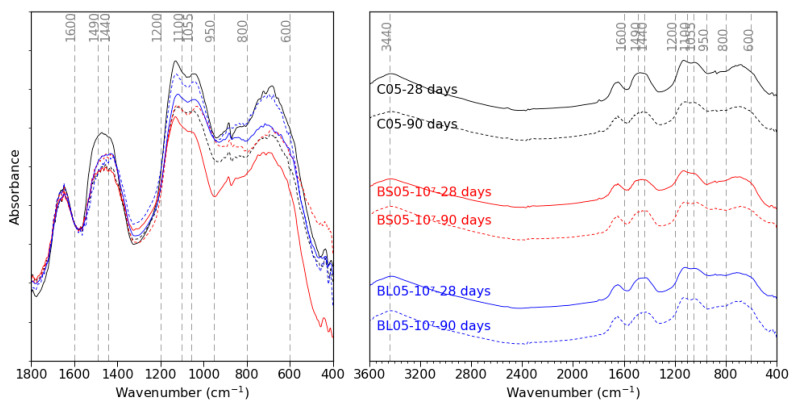
FTIR for 0.5 water–cement ratio mixes cured in sulfate. Black lines represent control mixes; red lines represent mixes with *B. subtilis*; blue lines represent mixes with *B. licheniformis*. Continuous lines represent mixes at 28 days; dashed lines represent mixes at 90 days.

Figure 10 and Figure 11 show the FTIR spectra of the mixes cured in water and sodium sulfate, respectively. On the left is the main phase zone with the curves superimposed at a wavelength of 1600 cm^−1^ to match the amplitudes of the spectra, and on the right is the total of the curves. For the analysis, the main phases were considered to complement the TGA: gypsum at ~600, ~680, and ~1100 cm^−1^, sulfates (SO_4_^2−^) at ~1100 and ~1200 cm^−1^, quartz at ~800 cm^−1^, water at ~1600 cm^−1^, carbonates (CO_3_^2−^) at ~1440 and ~1490 cm^−1^, portlandite (CH) at ~3643 cm^−1^ and contributions around ~1440–1460 cm^−1^, and C–S–H gel at ~950 and ~1055 cm^−1^. As can be seen, at 28 days, pastes with *B. subtilis* and *B. licheniformis* show relative increases in the normalized intensity of the C–S–H bands compared with the control mix. In particular, *B. subtilis* shows increases of around 5–6% at 950 and 1055 cm^−1^, while *B. licheniformis* reaches increases of close to 8% at 1055 cm^−1^. From a strictly spectral point of view, these results indicate a higher relative contribution of Si–O–Si vibrations associated with the C–S–H gel with respect to the water reference. In the carbonate region, the changes are more moderate and not uniform, showing that *B. subtilis* increases slightly by 1440 cm^−1^ (+1%) accompanied by a decrease by 1490 cm^−1^ (−1%), while *B. licheniformis* shows positive increases at both positions, although these are less than 3%. This asymmetry between 1440 and 1490 cm^−1^ suggests modifications in the relative distribution of CO_3_^2−^/CH contributions within the region rather than a uniform increase in carbonates. In the case of CH, the band at 3643 cm^−1^ increases marginally by 2% in *B. subtilis* and 1% in *B. licheniformis* at 28 days. At 90 days of water curing, spectral differences tend to reduce. In *B. subtilis*, the C–S–H bands practically converge to the control values, while *B. licheniformis* shows a slight relative decrease of −2% by 1055 cm^−1^. The normalized intensity of CH decreases by 2% in *B. subtilis*, while it remains close to the control in *B. licheniformis*. In terms of spectral form, an analysis of the area/amplitude ratio of the bands indicates an 8–13% reduction in the 28-day C–S–H region, particularly in *B. licheniformis*, which is consistent with relatively more concentrated bands—this effect is attenuated after 90 days.

In the sulfated environment, the relative differences with respect to the control are more significant, especially at early ages. At 28 days, *B. subtilis* shows decreases in normalized intensity in most of the analyzed bands: about −7% in C–S–H (950–1055 cm^−1^), −4% in carbonates (1440–1490 cm^−1^), −6 to −8% in gypsum (600 and 680 cm^−1^), and comparable reductions in sulfates (1100–1200 cm^−1^). Meanwhile, *B. licheniformis* in sulfate at 28 days shows much more limited variations with respect to the control, with differences generally less than ±3% both for C–S–H and for carbonates and sulfates. At 90 days of sulfate curing, both bacteria show positive increases in several regions. In *B. subtilis*, increases in the order of 3–4% are observed in CH (3643 cm^−1^), C–S–H, and carbonates, along with moderate increases in gypsum and sulfate bands. *B. licheniformis* at 28 days in sulfate shows much more limited variations with respect to the control, with differences generally less than ±3% in most bands both for C–S–H and for carbonates and sulfates.

### 3.7. Scanning Electron Microscopy

**Figure 12:**
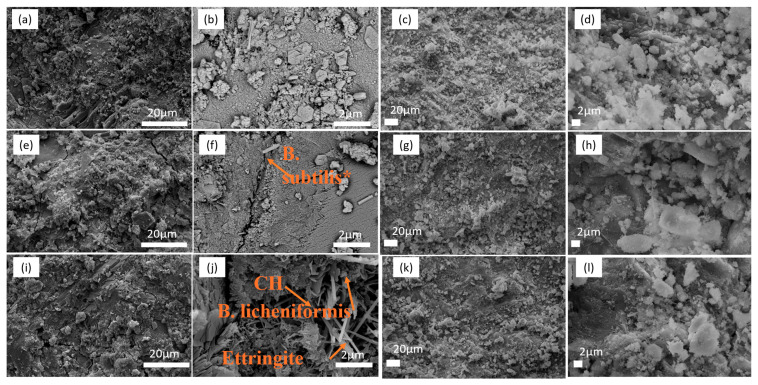
SEM of water-curing mixes, *w*/*c* = 0.5: (**a**,**b**) control at 28 days, (**c**,**d**) control at 90 days, (**e**,**f**) BS 10^7^ at 28 days, (**g**,**h**) BS 10^7^ at 90 days, (**i**,**j**) BL 10^7^ at 28 days, and (**k**,**l**) BL 10^7^ at 90 days. * Microstructure with *Bacillus* form.

**Figure 13:**
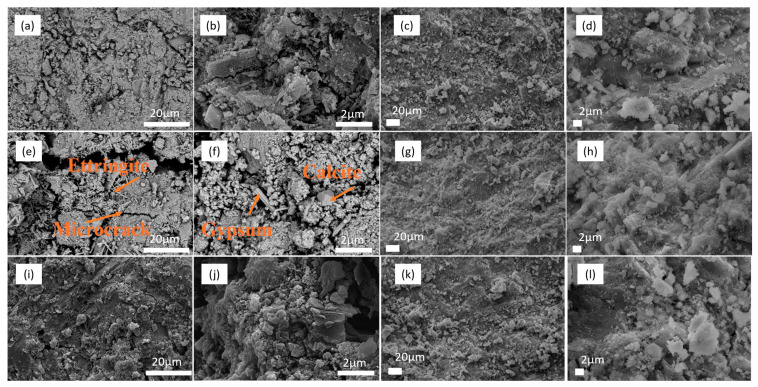
SEM of sulfate-curing mixes, *w*/*c* = 0.5: (**a**,**b**) control at 28 days, (**c**,**d**) control at 90 days, (**e**,**f**) BS 10^7^ at 28 days, (**g**,**h**) BS 10^7^ at 90 days, (**i**,**j**) BL 10^7^ at 28 days, and (**k**,**l**) BL 10^7^ at 90 days.

Figure 12 and Figure 13 show SEM images of the *w*/*c* = 0.5 mixes and the highest doses of bacteria used. During SEM analysis, water-cured mixes at 28 days showed regular-shaped microstructures with well-defined forms according to the literature, corresponding to calcium hydroxide, calcium carbonate ettringite, and amorphous phases, such as C–S–H. Mixes with bacteria, especially with *B. subtilis*, increased the amount of calcium carbonate and gypsum at 28 and 90 days. In the case of *B. licheniformis*, agglutinated spores were found in clusters and surrounded by ettringite and calcium hydroxide at 28 days (Figure 13). This could be because they are the first hydration products of cement paste, meaning that the bacteria added to the mix tend to adhere to them, and the sulfates could be creating a certain affinity that retains these spores. *B. subtilis* was not found in spore form, but it may have been found as a bacterium because it corresponds to the round-edged shape and the length and diameter of a filament shown in Figure 13d. As expected, mixes cured in sulfate had increased amounts of visible ettringite and gypsum, with more gypsum for *B. subtilis* than for *B. licheniformis*. However, amorphous phases presented more irregular forms, disaggregated in less continuous clusters—they became more consolidated at 90 days, hindering the observation of ettringite and gypsum. In this case, *B. subtilis* presented more ettringite than *B. licheniformis* both in water and in sulfates. Moreover, unlike calcium hydroxide, ettringite was covered by amorphous elements.

## 4. Discussion

Based on the results, it can be concluded that *B. subtilis* and *B. licheniformis* significantly influence the performance of cementitious mixtures subjected to external sulfate attack. In general, an increase in early-age compressive strength was observed in mixtures incorporating both bacteria when cured in water. Additionally, *B. licheniformis* initially delayed strength development in mixtures with high *w*/*c* ratios; however, this effect was progressively compensated over time, resulting in superior long-term performance. Previous studies showed consistent improvements in strength at early ages with normal curing. Typically, the literature reports strength increases of around 10 to 25% at ages of approximately 28 to 56 days [64,65,66,67,68]. In these cases, authors attribute improvements to CaCO_3_ mineralization from bacteria metabolism, issuing warnings if high bacteria dosages are used. However, little information was provided at later ages, or under ESA conditions. In this case, favorable strength changes were observed at later ages and under sulfate exposure, without significant variations in permeability. This contrasts to other authors who reported lower absorption, sorptivity, and penetration of chlorides due to bacterial action [66,67,69,70]. This would indicate that the observed improvements in durability are primarily associated with chemically driven modifications of the cement matrix induced by bacterial metabolism, rather than with physical pore blocking. This finding must be carefully addressed, because it could be related to limitations of an indirect measurement method, such as electrical resistivity, which does not directly measure hydraulic flow, but rather ionic connectivity of the porous solution. It may also be related to the ability of *B. licheniformis* to reduce sulfate expansion, since ESA depends on sulfate transport, ettringite/gypsum formation, and matrix ability to accommodate expansive products instead of pore blocking [8,70,71,72,73]. Therefore, it would suggest that *B. licheniformis* biochemically controls the reaction with sulfates and that it is not only the ingress of ions that is strengthened.

According to the microstructural and chemical modifications evidenced through FTIR, the intensity of C–S–H-related bands at ~950–1055 cm^−1^ was increased, indicating that *B. subtilis* and *B. licheniformis* enhanced the hydration and densification of the cementitious matrix. Similarly, TGA results showed higher mass losses in the 600–800 °C range associated with carbonate decomposition, confirming the formation of calcium carbonate through bacterial activity. These observations are consistent with previous studies on bacterial concrete, where bacteria mechanisms were associated with increased calcite precipitation, pore refinement, and mechanical enhancement [64,65,74]. However, unlike most of the recent literature, the present results demonstrate that overexpression of the calcium precipitation mechanism may lead to counterproductive effects under sulfate exposure in the long term, challenging the widespread assumption that calcium precipitation universally enhances durability through permeability reduction and crack sealing. In addition, in the present study, this adverse effect was mainly observed in mixtures containing *B. subtilis*, where calcium precipitation indirectly promoted the formation of gypsum and ettringite under sulfated curing conditions, thereby increasing susceptibility to expansion and long-term mechanical degradation. XRD analyses showed that under sodium sulfate exposure, ettringite-related peaks and gypsum-associated phases in *B. subtilis* mixtures increased by approximately 15% and 6%, respectively, compared with the control condition. FTIR spectra further supported this behavior, demonstrating higher intensities in sulfate-related bands within the 1100–1200 cm^−1^ region, together with moderate increases in carbonate-associated bands.

These results suggest that although *B. subtilis* enhanced carbonate precipitation and early matrix densification, the additional calcium availability may also have favored sulfate-driven expansive reactions. Similar studies commonly report beneficial effects of bacterial calcite precipitation but rarely evaluate whether excessive carbonate formation can unintentionally intensify AFt or gypsum formation during ESA [66,75]. In contrast, *B. licheniformis* exhibited markedly different behavior, characterized by a substantial reduction in expansion together with a sustained increase in compressive strength over time in specimens cured in sodium sulfate. This improved performance was consistent with the reduced presence of expansive phases identified by XRD and the attenuation of sulfate-related bands (1100–1200 cm^−1^) observed in FTIR spectra. Unlike *B. subtilis*, *B. licheniformis* showed reductions of approximately 7% in ettringite-associated phases and around 4% in gypsum formation under sulfate exposure, while maintaining relatively stable carbonate-related signals. TGA/DTG analyses additionally revealed that although carbonate decomposition remained significant, the relative intensities associated with sulfate-bearing phases were lower, indicating a more chemically stable matrix. The thermal decomposition profiles between 80 and 150 °C, commonly associated with AFt and gypsum dehydration, were notably less pronounced in *B. licheniformis* mixtures compared with *B. subtilis*. According to [76], sulfuric acid attack causes severe destabilization of the cement matrix, including decomposition of CH, reduction of C–S–H crystallinity, and formation of gypsum and ettringite detected through both FTIR and XRD. These authors associated these changes with loss of matrix cohesion and progressive microstructural deterioration.

Therefore, these microstructural changes cannot be directly interpreted as linearly proportional to the observed strength increase or expansion reduction. In terms of compressive strength, the literature consistently indicates that pore refinement and sealing associated with calcium carbonate precipitation are among the main mechanisms responsible for mechanical improvement. However, regarding sulfate-induced expansion, the present results suggest that deterioration depends more strongly on the reactivity and availability of phases, such as CH, CaCO_3_, and AFt, to interact with external sulfates than on permeability reduction alone. In this context, the behavior of *B. licheniformis* appears to differ from that of *B. subtilis*, by influencing the evolution and stability of sulfate-related reactions within the microstructure.

Therefore, the reduction in ettringite (~7%) and gypsum (~4%) in *B. licheniformis* mixtures is interpreted as one indicator of a modified sulfate reaction pathway, not as the sole quantitative cause of the observed expansion and strength changes. The performance improvement likely results from the combined effect of reduced sulfate-bearing phase formation, preserved C–S–H-related FTIR bands, carbonate-related TGA response, and reduced development of expansive products in confined pores. Similar limitations have been discussed in studies where sulfate damage was linked to mineral fronts, crystallization location, porosity, and mechanical degradation rather than simple bulk phase abundance [8,72,77,78].

Aygörmez and Canpolat [79] further demonstrated that long-term sulfuric acid exposure increases sulfate-related FTIR absorption bands while simultaneously weakening hydration product signatures, indicating chemical degradation rather than densification. Therefore, results suggest a low probability of the DSR metabolic pathway. Furthermore, Refs. [80,81,82] studied SRB, finding that DSR activity is performed under anaerobic conditions, which cannot be accomplished during curing. So, results indicate that the mixes’ improvement cannot be interpreted solely as a pore-filling mechanism, but rather as a coupled chemo-microstructural modification capable of altering the progression of external sulfate attack, where the ASR pathway stands as the most probable cause.

In comparison with classical SCMs, the results show similar improvements to silica fume and nanosilica under sulfate exposure. Reviews on nanosilica-modified systems indicate compressive strength increases typically up to 23% and sulfate expansion reductions of approximately 90%, mainly associated with pore refinement and CH consumption [83]. Similarly, silica fume-based mixtures generally report long-term strength gains of 15–40% and reduced gypsum/ettringite formation due to matrix densification [84]. *B. licheniformis*, which reached a general better performance, achieved strength increases approaching 90% under sulfate curing and expansion reductions near 80%, suggesting a response beyond conventional physical densification mechanisms. However, unlike SCMs, sulfate mitigation was not linked to microstructure densification by transformation of CH to CSH [85,86], instead suggesting a bacteria mechanism involving biochemical interactions with the cement paste that are capable of modifying sulfate reactions and limiting the accumulation of expansive phases. 

## 5. Conclusions

This research addressed the use of bacteria in cement paste mixes under ESA, using *B. subtilis* and *B. licheniformis* at different concentrations. The results show that these bacteria have a significant and variable impact on the mixes’ performance, leading to the following conclusions.

The application of *B. subtilis* and *B. licheniformis* can result in compressive strength improvements both under normal conditions and in the presence of external sulfates at low *w*/*c* ratios due to calcium precipitation. The effects improve over time, but complexities, such as a lack of heterogeneity, generate data dispersion. Likewise, overconcentration of bacteria often has negative effects on the mixes.

*B. subtilis* reduces the strength of cement pastes exposed to sodium sulfate, possibly due to the interaction between precipitated calcium carbonate and sulfates, which promotes gypsum reforming.

*B. licheniformis* can delay strength increases in high *w*/*c* mixes; however, it achieves better results in the long term.

The application of *B. licheniformis* markedly reduces the expansion of mortar exposed to sodium sulfate; however, an inverse relationship is seen between concentration and *w*/*c* ratio. *B. subtilis* does not generate significant benefits in terms of expansion.

When assessing permeability in terms of surface resistivity, it does not indicate a correlation with expansion results; that is, the effect of bacteria could be explained from a chemical point of view instead of pore sealing. As a result, it is important to limit calcium precipitation strategies for increasing durability in the presence of external sulfate due to the possibility of increasing gypsum and ettringite formation.

The microstructural changes observed in *B. licheniformis* differed from the degradation patterns commonly reported for sulfuric acid attack in cementitious materials. Considering the predominantly aerobic curing conditions of the specimens, the DSR pathway seems less likely. Therefore, the results suggest that *B. licheniformis* may interact with sulfate species through the ASR pathway or through other metabolic mechanisms capable of modifying the interaction between external sulfates and the cement paste microstructure.

The expression of calcium precipitation metabolism by bacteria increases the probability of calcium carbonate dissolution in continuous curing; therefore, care must be taken with the dosage to avoid loss of strength in the long term.

Despite the fact that *B. subtilis* is one of the most cited bacteria for self-healing and permeability control, its use should be carefully controlled to avoid ESA issues at later ages. *B. licheniformis* presents a mechanism based on its metabolic pathways to control ESA instead of pure pore sealing.

Although the strength and characterization results associated with the use of bacteria suggest a chemo-mechanical interaction between the microorganisms and the cementitious matrix, these findings must be interpreted and extrapolated with caution. Therefore, further research is still required to better understand mechanisms beyond calcium precipitation in concrete. While this represents a complex field of study, it also opens a promising pathway for the investigation and design of new bio-based strategies aimed at enhancing the performance and durability of cementitious materials.

This research has contributed significantly to advancing the understanding of bioconcretes. Future research may focus on survival mechanisms, bacteria agglutination effects, the combination of strains, reactions to other types of sulfates, and armor–bacteria interactions. Moreover, the metabolic pathways of *B. licheniformis* must be deeply studied regarding their direct action over sulfate-rich materials. From the results, it is expected that bioadditives could be designed as possible cement reducers to improve the concrete industry’s sustainability and productivity. In particular, potential applications in underground or coastal structures present high potential. Furthermore, potential applications for delayed ettringite formation could be addressed considering the response of *B. licheniformis* to sulfates and the resistance of spores to extreme conditions.

## Figures and Tables

**Table 1:** Literature review of bacteria concentration uses in concrete.

Concentration	Specie	Reference
10^3^ cells/mL 10^5^ cells/mL 10^7^ cells/mL	*B. subtilis*	Mondal and Ghosh (2018) [28]
10 × 10^5^ cfu/mL 50 × 10^5^ cfu/mL	*B. megaterium*	Andalib et al. (2016) [37]
10^8^ cells/mL	*B. subtilis*	Yamasamit et al. (2023) [38]
10^7^ spore/mL	*B. pseudofirmus* *B. cohnii* *B. halodurans*	Ivaškė et al. (2023) [39]
10^7^ cells/mL	*B. cohnii*	Wangui et al. (2020) [40]
10^9^ spore/mL	*B. subtilis* *B. sphaericus* *B. megaterium*	I. Ahmad et al. (2025) [41]
10^5^ cells/mL 10^6^ cells/mL 10^7^ cells/mL 10^8^ cells/mL 10^9^ cells/mL	*B. subtilis*	Ospina García et al. (2025) [42]
10^7^ cells/mL	*B. subtilis*	Salmasi and Mostofinejad (2020) [43]
10^5^ cells/mL	*S. pasteurii*	Chahal et al. (2012) [36]
10^7^ cells/mL	*B. licheniformis*	Nezafat Tabalvandani et al. (2023) [44]
10^9^ cells/mL	*B. licheniformis*	Yoon et al. (2024) [45]
10^7^ cells/mL	*B. sphaericus*	Sahoo et al. (2016) [46]
10^6^ cells/mL	*S. pasteurii*	Balam et al. (2017) [47]
1.5 × 10^6^ cells/cm^3^ 1.5 × 10^7^ cells/cm^3^	*B. cohnii*	Dipendraditya and Singh (2023) [48]

**Table 2:** Cement XRF characterization.

	C_3_S (%)	C_2_S (%)	C_3_A (%)	C_4_AF (%)	SiO_2_ (%)	Al_2_O_3_ (%)	Fe_2_O_3_ (%)	CaO (%)	SO_3_ (%)
Melón Super	62.2	14.5	6.3	10	21.4	4.5	3.3	65.2	1.9
ASTM C150 Type I	45–60	15–30	5–12	6–10	19–23	3–6	2–5	60–67	1–3

**Table 3:** Experimental matrix.

No.		*w*/*c* Ratio	Bacteria	Concentration of Bacteria (cells/mL)	Water (kg/m^3^)	Cement Water (kg/m^3^)	Sand (kg/m^3^)	Viscocrete 6000 (kg/m^3^)
1	CP	0.3	Control 1	0	474	1579	0	4.74
	Mortar				206	696	1463	0.70
2	CP	0.5	Control 2	0	600	1200	0	1.20
	Mortar				261	529	1463	0.53
3	CP	0.3	BS	10^5^	474	1579	0	4.74
	Mortar				206	696	1463	0.70
4	CP	0.3	BS	10^6^	474	1579	0	4.74
5	CP	0.3	BS	10^7^	474	1579	0	4.74
	Mortar				206	696	1463	0.70
6	CP	0.5	BS	10^5^	600	1200	0	1.20
	Mortar				261	529	1463	0.53
7	CP	0.5	BS	10^6^	600	1200	0	1.20
8	CP	0.5	BS	10^7^	600	1200	0	1.20
	Mortar				261	529	1463	0.53
9	CP	0.3	BL	10^5^	474	1579	0	4.74
	Mortar				206	696	1463	0.70
10	CP	0.3	BL	10^6^	474	1579	0	4.74
11	CP	0.3	BL	10^7^	474	1579	0	4.74
	Mortar				206	696	1463	0.70
12	CP	0.5	BL	10^5^	600	1200	0	1.20
	Mortar				261	529	1463	0.53
13	CP	0.5	BL	10^6^	600	1200	0	1.20
14	CP	0.5	BL	10^7^	600	1200	0	1.20
	Mortar				261	529	1463	0.53

Note: CP, cement paste mix; BS, *Bacillus subtilis*; BL, *Bacillus licheniformis*.

**Table 4:** OLS model analysis mixes cured in water.

Variable	β BS	Significance OLS fc-BS	β BL	Significance OLS fc-BL	
*w*/*c*	−11.55	*p* < 0.001	−11.99	*p* < 0.001	Negative dominant variable. Higher *w*/*c* reduces strength.
C	31.18	*p* ≈ 0.06	−3.59	*p* > 0.8	Positive variable. Relevant for *B. subtilis*, but not determinative for *B. licheniformis*.
C^2^	−33.60	*p* < 0.05	3.58	*p* > 0.8	Evidence of bacterial overdosage of BS adversely affected; in BL it is positive, but of low impact.
T	19.73	*p* < 0.01	26.09	*p* < 0.001	Positive variable. Progressive development of strength due to hydration and bacterial activity. This effect is particularly relevant for BL.
T^2^	−16.51	*p* < 0.01	−21.62	*p* < 0.001	Negative variable. Deceleration or stabilization of endurance at long ages.

**Table 5:** OLS model analysis mixes cured in sulfate.

Variable	β BS	Significance OLS fc-BS	β BL	Significance OLS fc-BL	Description
*w*/*c*	−0.83	*p* < 0.001	−0.710	*p* < 0.001	Negative key control.
C	2.33	*p* ≈ 0.065	2.89	*p* = 0.024	Dose effect: relevant in BS (tendency to increase), not determinant in BL.
C^2^	−2.42	*p* ≈ 0.056	−2.861	*p* = 0.026	Dose nonlinearity: in BS there is evidence of overdosage (optimal), in BL it does not contribute.
T	−0.02	*p* > 0.5	0.326	*p* > 0.5	Gain resistant with age. Stronger in BL.
T^2^	0.01	*p* > 0.8	0.089	*p* > 0.8	Long-term slowdown/stabilization (negative temporal curvature).

**Table 6:** TGA analysis of water–cement ratio mixes cured in water.

	0–200 °C		200–400 °C		400–550 °C		600–850 °C		ΔCH Mass (%)	ΔCaCO_3_ Mass (%)
	T° Peak (°C)	Mass (%)	T° Peak (°C)	Mass (%)	T° Peak (°C)	Mass (%)	T° Peak (°C)	Mass (%)		
C05-28	113.66	94.79	200.00	89.38	469.86	81.62	671.87	78.61	−3.22	−0.88
C05-90	110.81	95.22	201.38	89.33	464.65	81.82	694.07	77.14	−2.90	−1.93
BS05-28	115.98	93.71	200.15	88.26	474.89	80.77	679.25	77.76	−3.08	−1.06
BS05-90	103.99	95.57	200.08	89.26	463.44	81.57	684.29	77.36	−2.71	−1.29
BL05-28	105.19	95.06	200.05	89.41	465.46	81.47	644.39	78.81	−3.59	−0.48
BL05-90	103.07	95.03	201.31	88.73	463.29	80.55	664.73	77.57	−3.43	−0.74

**Table 7:** TGA analysis of water–cement ratio mixes cured in sulfate.

	0–200 °C		200–400 °C		400–550 °C		600–850 °C		ΔCH Mass (%)	ΔCaCO_3_ Mass (%)
	T° Peak (°C)	Mass (%)	T° Peak (°C)	Mass (%)	T° Peak (°C)	Mass (%)	T° Peak (°C)	Mass (%)		
C05-28	110.09	95.12	200.12	89.58	469.10	82.07	681.90	78.37	−2.91	−1.28
C05-90	108.20	95.69	200.38	90.22	462.45	83.06	699.45	77.47	−2.50	−2.52
BS05-28	106.33	95.16	200.15	89.65	464.99	82.14	685.66	78.25	−3.19	−1.61
BS05-90	108.15	95.26	200.79	89.50	462.20	82.83	695.48	77.84	−2.24	−2.37
BL05-28	109.98	95.55	200.10	90.29	471.30	82.76	665.01	79.38	−3.11	−1.10
BL05-90	108.26	94.46	200.02	88.22	470.09	79.50	661.44	76.73	−3.61	−0.50

## Data Availability

The original contributions presented in the study are included in the article, further inquiries can be directed to the corresponding author.
